# Glucan exopolysaccharide from *Enterococcus hirae* OL616073 alleviates oxidative stress and extends lifespan in different model systems

**DOI:** 10.3389/fmolb.2026.1901988

**Published:** 2026-08-21

**Authors:** Uma Rani Potunuru, Padmapriya Ramamoorthy, Balasubramanian Chellammal Muthubharathi, Bargavi A. Meenachi, Raksha Goswami, Palanisamy Bruntha Devi, Anjali Mohan, Krishnaswamy Balamurugan, Arunkumar Dhayalan, Digambar Kavitake, Prathapkumar Halady Shetty

**Affiliations:** 1 Department of Food Science and Technology, Pondicherry University, Pondicherry, India; 2 Department of Biotechnology, Alagappa University, Karaikudi, Tamil Nadu, India; 3 Biochemistry and Molecular Biology, School of Medicine, University of New Mexico, Albuquerque, NM, United States; 4 Department of Biotechnology, Pondicherry University, Pondicherry, India; 5 Department of Biosciences, Manipal University Jaipur, Jaipur, Rajasthan, India; 6 Research and Development Laboratory, Fermiayur LLP Division, Mysore Mercantile Co. Ltd., Bangalore, India

**Keywords:** antioxidant potential, *Caenorhabditis elegans*, CaCo-2, *Enterococcus* hirae OL616073, glucan exopolysaccharide, reactive oxygen species

## Abstract

**Objective:**

This study aims to evaluate the oxidative stress alleviating potential of glucan exopolysaccharide (EPS) using *in vitro* (biochemical and cell culture) and *in vivo Caenorhabditis elegans* model systems.

**Methods:**

The *in vitro* antioxidant potential of EPS from *Enterococcus hirae* OL616073 of food origin was evaluated using reducing power, ferric-reducing antioxidant power (FRAP) assay, and 2,2-azino-bis (3-ethylbenzothiazoline-6-sulfonic acid) (ABTS) radical-scavenging activity assays. The antioxidant effect of EPS was also evaluated in *C. elegans* (wild type Bristol N2), a simple multi-cellular eukaryotic model via survival assay, gene expression, DCF-DA staining, reproduction, and pharyngeal pumping assay. Finally, the ROS scavenging activity of EPS was investigated in human colon cancer cells (Caco-2 cell line) by DCF-DA staining and flow cytometry.

**Results and discussion:**

The *in vitro* assays showed the significant antioxidant activity of EPS as evidenced by reducing power, FRAP, and ABTS radical-scavenging activity. In *C. elegans*, EPS treatment (150 and 300 μg/mL) significantly extended the lifespan of wild-type (Bristol N2) *C. elegans* (∼1.21% and ∼0.83%) compared to control. EPS treatment (150 μg/mL) resulted in increased egg-laying capacity and little change in the pharyngeal pumping rate of *C. elegans* over five consecutive days. In addition, pre-, post-, and co-treatment of EPS (150 μg/mL) in the presence or absence of H_2_O_2_ (1 mM) showed a reduction in ROS generation. Similarly, in the colorectal cancer cell line (Caco-2), high basal intracellular ROS levels were reduced with increasing concentration of EPS via an increase in intracellular antioxidant enzymes.

**Conclusion:**

EPS from *E. hirae* OL616073 of food origin exhibited significant antioxidant potential across diverse model systems. These findings highlight its potential as a natural antioxidant ingredient for applications in functional foods, nutraceuticals, and pharmaceutical formulations.

## Graphical Abstract

**Figure:**
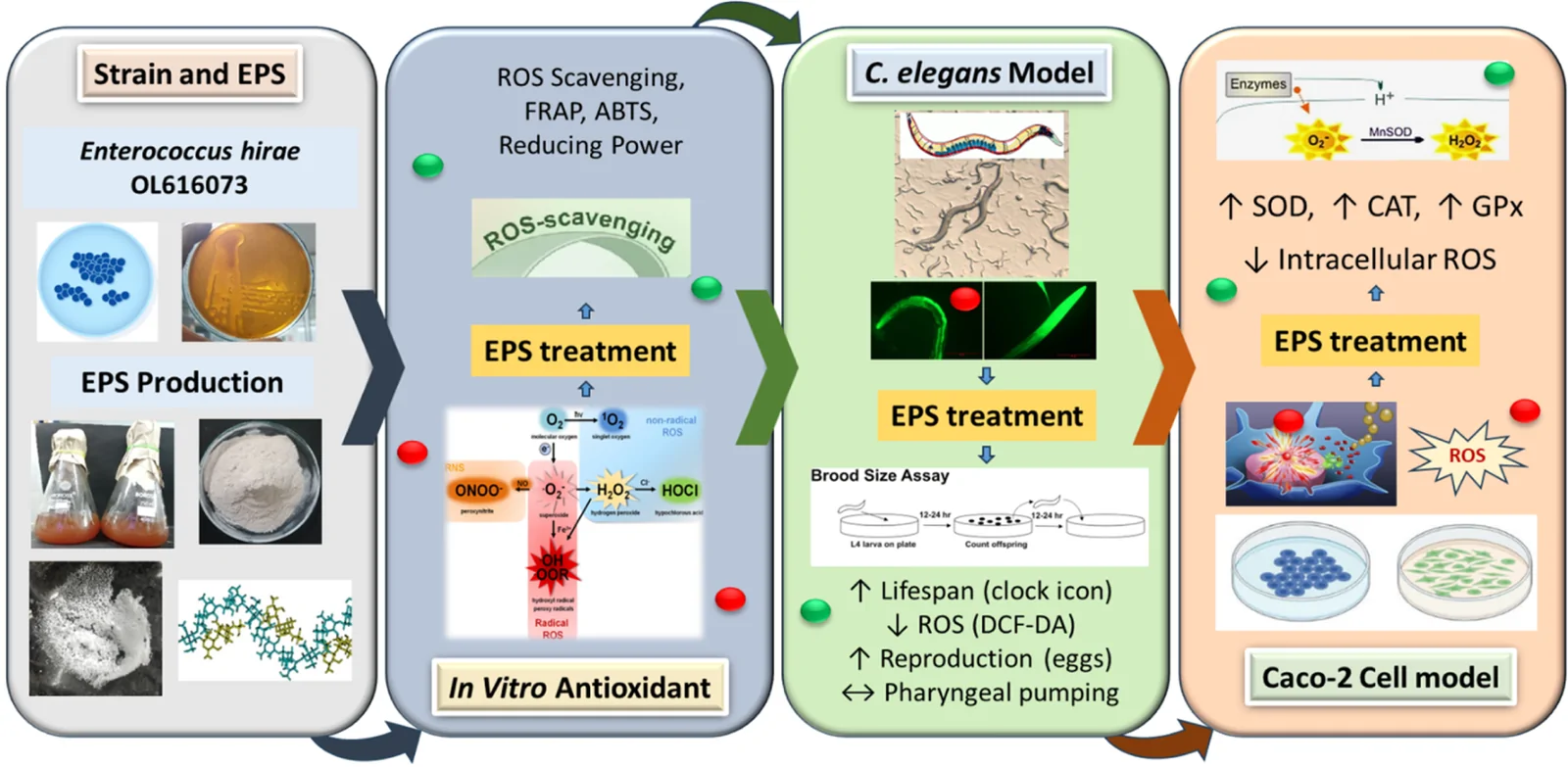

## 1 Introduction

Endogenous free radicals are generated in cells during oxidative reactions due to cellular metabolism and from exogenous sources such as exposure to radiation (X-rays, UV-rays, and γ-rays), harmful chemicals, heavy metals, high temperature, environmental pollutants, drugs, and microbial infections (Martemucci et al., 2022; Pham-Huy et al., 2008). Free radicals including reactive oxygen species (ROS) and reactive nitrogen species (RNS) are produced from oxygen and nitrogen molecules during normal metabolic processes (Phaniendra et al., 2015; Stief and Fareed, 2003; Valko et al., 2007). Though free radicals at physiological levels are not always detrimental, excessive free radicals are scavenged by the cellular antioxidant machinery (Chandimali et al., 2025). High levels above the threshold can deteriorate normal cellular activities by causing oxidative damage to cellular macromolecules such as DNA, proteins, and lipids, resulting in cellular disruption. Over time, this may result in mutations, chromosomal instability, and cellular degeneration, leading to severe health impairments, including metabolic diseases such as diabetes, cardiovascular and inflammatory diseases, cancer, asthma, and hepatic cirrhosis, and accelerated aging (Cho et al., 2022; Li et al., 2025; Muro et al., 2024; Shaito et al., 2022; Teleanu et al., 2022).

The cell’s antioxidant machinery delays and prevents oxidative damage due to free radicals, thus maintaining normal cellular functions (Martemucci et al., 2022; Tan et al., 2018). Counteracting the generation of endogenous and exogenous free radicals and neutralizing their accumulation by administering antioxidant-rich molecules through the diet can help efficiently manage oxidative-stress-induced cellular and systemic imbalance, thereby preventing chronic and degenerative diseases (Sharifi-Rad et al., 2020). Natural antioxidants derived from plant foods and microorganisms have gained prominence in recent years due to their strong free radical scavenging abilities and few safety concerns (Rani et al., 2021; Saeed et al., 2012). Antioxidants derived from microorganisms are considered to be sustainable and cost-effective alternatives in food and pharmaceutical applications (Chandra et al., 2020). Recent reports on the human gut microbiome have also indicated the role of these molecules in health and disease, with no adverse effect on gut homeostasis (Fan and Pedersen, 2021; Gomaa, 2020).

Microbial exopolysaccharides (EPSs) produced from lactic acid bacteria (LAB) strains have shown antioxidant properties (Garcia Mansilla et al., 2025; Khalil et al., 2022; Sharma et al., 2024). In addition to their antioxidant property, EPSs have been shown to possess potential anticancer, antibacterial, antiviral, immunomodulatory, gut-microbiota-modulatory, and cholesterol-lowering activities (Abdalla et al., 2021; Adebayo-Tayo et al., 2018; Bengoa et al., 2020; Khalil et al., 2022; Wu et al., 2021; Zhang et al., 2024).

EPSs isolated from various strains of *Enterococcus* spp. have also reported to have effective antioxidant activity (Abdhul et al., 2014; Choudhuri et al., 2020; Kavitake et al., 2023). For example, *Astragalus membranaceus* polysaccharides have been reported to prevent intestinal inflammation and oxidative stress injury in a type2 diabetes mouse model, modulating the gut microbiome by increasing beneficial gut bacteria and preventing intestinal pathogens (Chen et al., 2022). In a previous study from our group, galactan EPS produced by *Weissella confusa* KR780676 was reported to exhibit antioxidant activity by scavenging ROS (Kavitake et al., 2022). Our earlier study demonstrated the oxidative stress-alleviating effect of a glucan EPS isolated from *Leuconostoc lactis*, derived from an Indian traditional fermented batter (Saravanan et al., 2019). We recently published a complete characterization of glucan produced by *Enterococcus hirae* OL616073 of food origin (Kavitake et al., 2024). The *in vitro* prebiotic potential and gut microbial modifications (increase in Bacillota abundance) and short chain fatty acid (SCFA) production by the glucan are reported in previous studies from our research group to support its role in gut microbial resilience and energy metabolism in protecting the gut from potential injury due to infection and ROS damage (Tiwari et al., 2025). The current homopolysaccharide with abundant glucose units and highly branched porous structure with strong water solubility provides novelty to be exploited for a number of biological functions in cells and the whole organism. The current study aims to investigate the *in vitro* (biochemical and cell culture) and *in vivo* (*Caenorhabditis elegans*) antioxidant potential of this glucan EPS (hereafter, addressed as EPS). Additionally, we evaluated its anti-aging properties using the *C. elegans* model.

## 2 Materials and methods

### 2.1 Reagents

Wild-type *C. elegans* (Bristol N2) and *Escherichia coli* OP50 were obtained from the *Caenorhabditis* Genetics Center (CGC), University of Minnesota, United States. Dulbecco’s minimum essential medium (DMEM), De Man, Rogosa, and Sharpe (MRS) broth (Cat no. GM369), antibiotic-antimycotic solution, trypsin-EDTA, fetal bovine serum (FBS), acridine orange, ethidium bromide, NGM (nematode growth medium, prepared with 0.5% peptone, 1% NaCl, and 1.8% agar, following sterilization at 121 °C for 20 min, 0.025% of 1 M of KPO_4_, 0.001% of 1 M of CaCl_2_, 0.001% of 1 M of MgSO_4_, 0.001% of cholesterol dissolved in 95% ethanol, 0.0001% of streptomycin, and nystatin), bacteriological agar, peptone, tryptone, phosphate-buffered saline (PBS)-1X (Cat no. TL1006), ABTS (2,2-azino-bis(3-ethylbenzothiazoline-6-sulfonic acid); Cat no. MB255), and 2,4,6-tris(2-pyridyl)-s-triazine (TPTZ) (Cat no. RM1487) were purchased from HiMedia Thane (West), Maharashtra, India. We purchased 2′,7′-dichlorofluorescein diacetate (DCF-DA) (Cat no. D6883) and 5′-fluoro-5-deoxyuridine (Cat no. F8791) from Sigma-Aldrich, United States and RNeasy Mini Kit from QIAGEN (Cat no. 74104), TRIzol reagent from Ambion (Cat no. 15596026), Verso cDNA synthesis Kit (Thermo Scientific Cat no. AB1453A), catalase colorimetric activity kit (Invitrogen, EIACATC 192 tests), Fast Start SYBR Green Master (Roche Cat no. 06402712001), and oligonucleotide sequences from Eurofins. Caco-2 (colon cancer cell line) was procured from the National Center for Cell Science, Pune, India.

### 2.2 Preparation of glucan EPS

The EPS producing strain, *E. hirae* OL616073, was isolated from fermented *idli* batter. The production and purification of EPS was carried out as per Kavitake et al. (2024). Briefly, the culture was incubated for 60 h at 30 °C in MRS broth with 2% sucrose added under static conditions. The cell suspension was centrifuged at 12,000 ×*g* for 15 min to collect biomass. Proteins were precipitated by adding trichloroacetic acid solution (2% w/v) to the supernatant and separated by centrifugation (12,000 ×*g*) at 4 °C for 30 min. Chilled ethanol in a 1:3 ratio was used overnight to precipitate EPS. After 15 min of centrifugation at 19,200 ×*g*, the crude precipitate was dissolved in Milli-Q water and freeze-dried. EPS was further purified by ion-exchange chromatography as follows: a crude precipitate of EPS was transferred to a dialysis bag (12–14 kDa) and dialyzed for 48 h at 4 °C using Milli-Q water. In the purification phase, a 50 mg/mL EPS sample was first dissolved in deionized water and then centrifuged for 15 min at 15,000 × g. The supernatant was collected and subjected to anion exchange chromatography using a DEAE Cellulose-52 column (16 mm × 20 cm) to eliminate any negatively charged protein and nucleic acid contaminants from the EPS solution. Major fractions containing polysaccharides were pooled, dialyzed against water, and lyophilized. The lyophilized EPS was used for the cell culture experiment.

### 2.3 Biochemical assays for screening antioxidant properties of EPS

#### 2.3.1 Reducing activity

The reducing activity of different concentrations of EPS was investigated according to a method suggested by Das and Goyal (2015). The sample or distilled water as control (0.2 mL) was mixed with 1% potassium ferricyanide (0.2 mL) and 20 mmol/L sodium phosphate buffer (0.2 mL), pH 7. The mixture was incubated in a water bath at 50 °C for 20 min. Later, 10% (w/v) trichloroacetic acid (0.2 mL) was added to terminate the reaction. The solution was centrifuged at 780 *g* at 4 °C for 10 min. The supernatant (0.5 mL) was mixed with 0.1% (w/v) ferric chloride (0.1 mL) and distilled water (0.4 mL). Absorbance was measured at 700 nm. The reducing power of EPS was expressed as μg/mg of ascorbic acid equivalent (AAE/mg).

#### 2.3.2 Ferric-reducing antioxidant power

Ferric-reducing antioxidant power (FRAP) of EPS was evaluated at different concentrations. The FRAP reagent was prepared by mixing 0.3 M acetate buffer (pH 3.6), 0.01 M 2,4,6-tris(2-pyridyl)-s-triazine (TPTZ) in 0.04 M HCl, and 0.02 M FeCl_3_ in a 10:1:1 proportion. A 0.3 mL sample was added to 2.7 mL of freshly prepared FRAP reagent. The mixture was incubated for 10 min at 37 °C, and absorbance was recorded at 593 nm. Ferrous sulfate was used to plot a linear calibration curve, and the result was expressed as micromoles of ferrous equivalents per mg of EPS (μmol Fe(II)/mg). Ascorbic acid was used as a positive control (Bomfim et al., 2020).

#### 2.3.3 ABTS assay

ABTS (2,2-azino-bis (3-ethylbenzothiazoline-6-sulfonic acid)) radical scavenging assay was used to study the antioxidant property of EPS. We mixed 5 mL of 7 mM ABTS with 88 µL of 140 mM potassium persulfate to prepare the ABTS reagent, which was stored for 12–16 h in the dark at room temperature to produce ABTS + cation radicals. After incubation, the reagent was diluted with water till it reached an OD about 0.7–0.73 at 734 nm. ABTS solution (100 µL) was added to different concentrations (100 µL) of EPS. The mixture was incubated at room temperature for 6 min, and absorbance was measured at 734 nm (Lee et al., 2015). Quercetin was used as a positive control. To determine the ABTS scavenging activity of EPS, the following formula was used.

### 2.4 Evaluation of oxidative stress alleviation in *Caenorhabditis elegans* model

#### 2.4.1 Survival assay

Wild-type (Bristol N2) *C. elegans* was maintained in Nematode Growth Medium (NGM) seeded with *E. coli* OP50 (Uracil auxotroph and food source for *C. elegans*) 4366476 (Brenner, 1974). The worms were maintained at 20 °C. The synchronized population of L4 worms was taken for all the experiments. Synchronization was obtained by lysing the gravid worms with an alkaline sodium hypochlorite solution containing 5 M potassium hydroxide (KOH) in a 1:1 ratio (Sivamaruthi and Balamurugan, 2014). *E. coli* OP50 was maintained in a Luria–Bertani medium at 37 °C. For experiments, bacterial cultures of 0.2 OD at 600 nm were used (Muthubharathi et al., 2021). For all the experiments, stage synchronized L4 worms were used in triplicate.

The survival assay was performed as per Pooranachithra et al. (2021). The experiment was carried out in 24 well plates, to which 20% of 0.2 OD at 600 nm of *E. coli* OP50 was added. The impact of EPS on the lifespan of *C. elegans* was tested by providing the compound in concentrations of 100, 150, 200, 250, 300, 450, and 600 μg/mL to the respective assay wells. Observations were made regularly using a stereo microscope (Nikon SMZ1000, Japan). The graph was plotted using OriginPro 2018, and the bar represents standard error.

#### 2.4.2 Reproduction and pharyngeal pumping assay

The assay was performed as per Muthubharathi et al. (2021). The experiment was performed in 10 mm-sized solid NGM media. The plates were either lawn-formed with *E. coli* OP50 (100 µL of 0.2 OD culture), EPS (100 µL from 1 mg/mL stock), or EPS along with *E. coli* OP50. Each plate carried six specimens of *C. elegans*. The worms were regularly transferred to a new plate to avoid confusion between parents and progeny. Observations were made using a stereo microscope (Nikon SMZ1000, Japan). The eggs laid the number of worms were counted and cross-verified the next day by counting the L1 progeny. Similarly, the pumping rate of the pharynx was observed manually for 30 s for each worm.

#### 2.4.3 ROS detection by DCF-DA

Control or untreated, 1 mM of H_2_O_2_-treated (either 30 min or 2 h) and EPS-treated (2 h) samples were collected in a fresh Eppendorf tube from the assay plate by washing them with M9 buffer. The supernatant was removed completely. The worms were again washed with M9 buffer three times. Then, 500 µL of M9 buffer was added after washing. To the sample, 50 µL of 100 µM of DCF-DA was added. The samples were mixed by gentle tarring. They was incubated in the dark for 15–20 min. After incubation, the supernatant was removed. Then the pellet (worms) was washed with 1X PBS and again with M9 buffer (Kamaladevi et al., 2016). After removing the supernatant, 100 µL of M9 buffer was added. To the solution (along with worms in the pellet), 10 mM of sodium azide was added. After incubation for 2–3 min, the worms were pipetted out, placed on a glass slide, and subsequently imaged under a fluorescence microscope (Nikon ECLIPSE Ts2R, Japan).

### 2.5 Assessment of oxidative stress alleviation in the mammalian cell model

#### 2.5.1 ROS detection in Caco-2 cells by DCF-DA staining and flow cytometry

A cell-permeable fluorescent dye, DCF-DAis oxidized to fluorescent DCF by the intracellular ROS such as H_2_O_2_. The intensity of fluorescence is proportional to the amount of intracellular ROS generation. Increase in oxidative stress is indicated by an increase in relative ROS levels. Flow cytometry was used to measure the fluorescence intensity of DCF-DA-stain in Caco-2 cells by a modified method as per Morresi et al. (2019).

Briefly, log-phase Caco-2 cells (2 × 10^5^) maintained in DMEM were pre-treated with three different concentrations of EPS (1, 5, and 10 mg/mL) for 24 h. Following treatment, the cells were incubated with 2.5 µM DCF-DA solution (prepared in basal DMEM) for 45 min. After incubation, they were subject to mild trypsinization to make a cell suspension, followed by washing with 1X PBS, and single-cell suspensions were made with basal DMEM media for flow cytometry analysis. A range of EPS concentrations (0.625, 1.25, 2.5,5,10, and 20 mg/mL) were tested for cytotoxicity in Caco-2 using the MTT cytotoxicity assay; they were found to have no effect on cell viability up to 20 mg/mL.

#### 2.5.2 Antioxidant biomarker expression in Caco-2

The major enzymatic and non-enzymatic intracellular antioxidant defense systems are superoxide dismutase (SOD), catalase (CAT), glutathione peroxidase (GPX), glutathione S-transferase (GSH), and glutathione.

#### 2.5.3 Antioxidant gene expression by real time PCR (RT-PCR)

The effect of EPS treatment on the expression of CAT, GPX, and SOD genes was quantified in Caco-2 cells by using quantitative real-time PCR (qRT-PCR) as per Awasthi et al. (2018) and Kumar et al. (2020). Briefly, total RNA isolation and reverse transcription was performed by using TRIzol reagent (Ambion) and Maxima H Minus Reverse Transcriptase (Thermo Scientific), respectively, as per the manufacturer’s instructions. The qRT-PCR analysis was carried out in a Light Cycler Real-Time PCR system (Roche) using Fast Start Essential Green Master Mix (Roche). The sequences of primers that were utilized in the qRT-PCR are provided in Table 1.

**TABLE 1:** Details of primers.

Sl no.	Name of gene	Species	Forward primer	Reverse primer
1	SOD 1	Human	GTGAAGGTGTGGGGAAGCAT	AAGTCTCCAACATGCCTCTCTT
2	CATA	Human	GAAGATGCGGCGAGACTTTC	CCTTGTGAGGCCAAACCTTG
3	GSHPRX	Human	GCGGGGCAAGGTACTACTTA	GTTCTTGGCGTTCTCCTGATG
4	GAPDH	Human	TCACCAGGGCTGCTTTTAAC	TGACGGTGCCATGGAATTTG

The qRT-PCR experiments were performed in triplicate and in three different biological replicates. The expression of these genes was normalized to the expression of the glyceraldehyde-3-phosphate dehydrogenase (GAPDH) and presented as relative values to the control cells, which were not treated with EPS.

### 2.6 Statistical analysis

The results are represented as the mean ± standard deviation (SD). Error bars indicate the SD of results unless specified otherwise, obtained from at least three independent experiments. For statistical analysis, two-way analysis of variance (ANOVA) followed by Tukey’s test was performed using GraphPad Prism 6.01, unless specified otherwise. *p* < 0.05 is considered significant.

## 3 Results

### 3.1 *In vitro* antioxidant activity

#### 3.1.1 Reducing power assay

**FIGURE 1:**
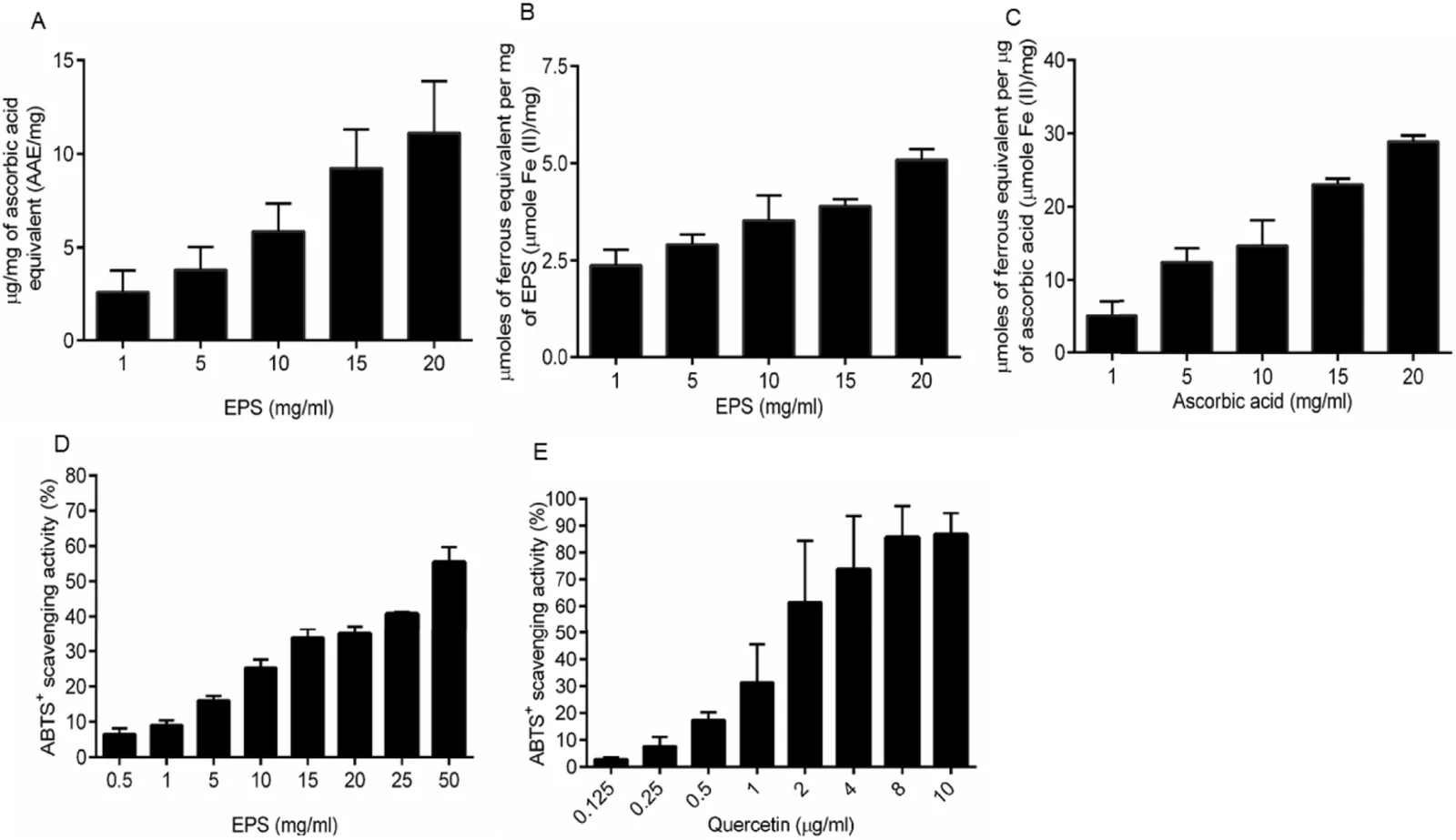
Reducing power, FRAP assay, and ABTS radical scavenging activity of EPS. The reducing activity **(A)** was expressed as μg/mg of ascorbic acid equivalent (AAE/mg). The antioxidant power of EPS **(B)** and ascorbic acid **(C)** was evaluated based on a calibration curve of FeSO_4_ and expressed in µmoles of ferrous equivalent per mg of EPS. The percentage ABTS radical scavenging of EPS **(D)** and quercetin **(E)** was determined for a range of concentrations. Values are expressed as the mean ± SEM of three independent experiments.

The reducing power assay was used to evaluate the antioxidant property of glucan EPS. The ability of EPS to reduce Fe^3+^ to Fe^2+^ correlates directly with its antioxidant capacity. The reducing activity of EPS was determined and expressed as μg/mg of ascorbic acid equivalent (AAE/mg). The concentration ranges of a minimum of 1 mg/mL to a maximum of 20 mg/mL of EPS were used to evaluate antioxidant activity. The reducing power of glucan EPS at the concentration of 1 mg/mL was 2.627 ± 0.666 μg of ascorbic acid equivalents (2.627 ± 0.666 μg AAE/mg) and 20 mg/mL was 11.117 ± 1.596 11.117 ± 1.596 μg AAE/mg. Thus, reducing power was found to increase with increasing concentrations of EPS (Figure 1A).

#### 3.1.2 FRAP assay

The ferric-reducing antioxidant power assay is a direct method for measuring the antioxidant capacity of EPS based on its ability to reduce the ferric tripyridyltriazine to the ferrous tripyridyltriazine (Fe^2+^, TPTZ) ion. The reducing activity was related to their electron-donating ability to scavenge free radicals. In this assay, the antioxidant power of EPS was evaluated based on a calibration curve of FeSO_4_. The antioxidant activity of EPS and ascorbic acid was expressed as micromoles of ferrous equivalents. The concentration ranged from a minimum of 1 mg/mL to a maximum of 20 mg/mL to evaluate antioxidant property. The ferric-reducing activity of glucan EPS at concentrations of 1 and 20 mg/mL showed 2.378 ± 0.400 and 5.090 ± 0.272 μmol Fe(II)/mg μmol of ferrous equivalents, respectively. Ferric-reducing antioxidant power of different concentrations of EPS and ascorbic acid is shown in Figures 1B and C, respectively.

#### 3.1.3 ABTS radical scavenging activity

The ABTS assay is used to measure the antioxidant property of EPS by determining its ability to scavenge ABTS radicals. ABTS+ is a stable synthetic free radical which is used to estimate the total antioxidant power of a compound. The ABTS radical scavenging effect of EPS is represented within a range of 0.5–50 mg/mL of EPS. According to the results, 0.5 mg/mL and 50 mg/mL concentrations of EPS showed 6.550% ± 1.629% and 55.451% ± 4.248% ABTS + radical scavenging activity, respectively. The ABTS + radical scavenging activity of different concentrations of EPS and positive control quercetin is shown in Figures 1D and E.

### 3.2 EPS reduced oxidative stress in *Caenorhabditis elegans*

#### 3.2.1 EPS moderately enhanced the lifespan of *C. elegans*

**FIGURE 2:**
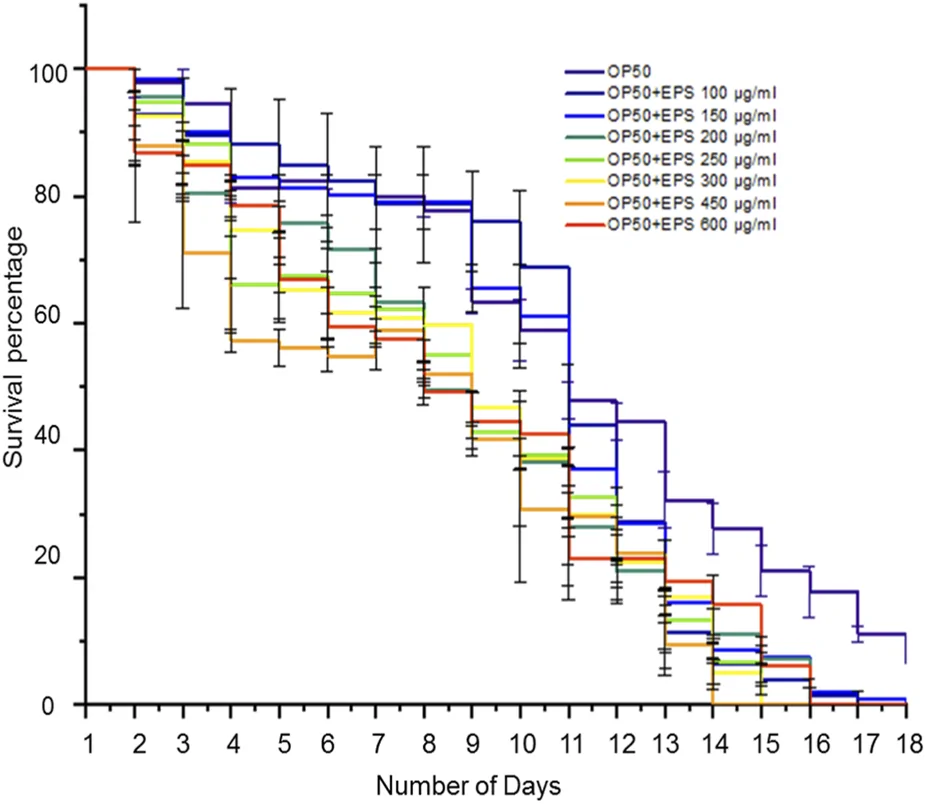
Survival assay of *Caenorhabditis elegans* upon EPS co-supplementation with *E. coli* OP50 for 18 days.

The impact of EPS on *C. elegans* was studied by survival assay, and thereby the minimal dosage that enhanced lifespan was determined. EPS was added along with *E. coli* OP50, and the survival of *C. elegans* was assessed regularly. The observations showed that, at 150 and 300 μg/mL of EPS, the survival of *C. elegans* was improved to ∼1.21% and ∼0.83% compared to control (Figure 2). Hence, it is suggested that, at the given concentration, a moderate extension of lifespan occurred in *C. elegans,* mainly due to the protection against oxidative stress mediated aging by the EPS. Therefore, 150 μg/mL (15% of supplement) of EPS is considered the minimal required concentration for *C. elegans*. The same concentration (150 μg/mL) was used to study the physiological and biochemical studies.

#### 3.2.2 EPS exposure positively enhanced vital parameters

**FIGURE 3:**
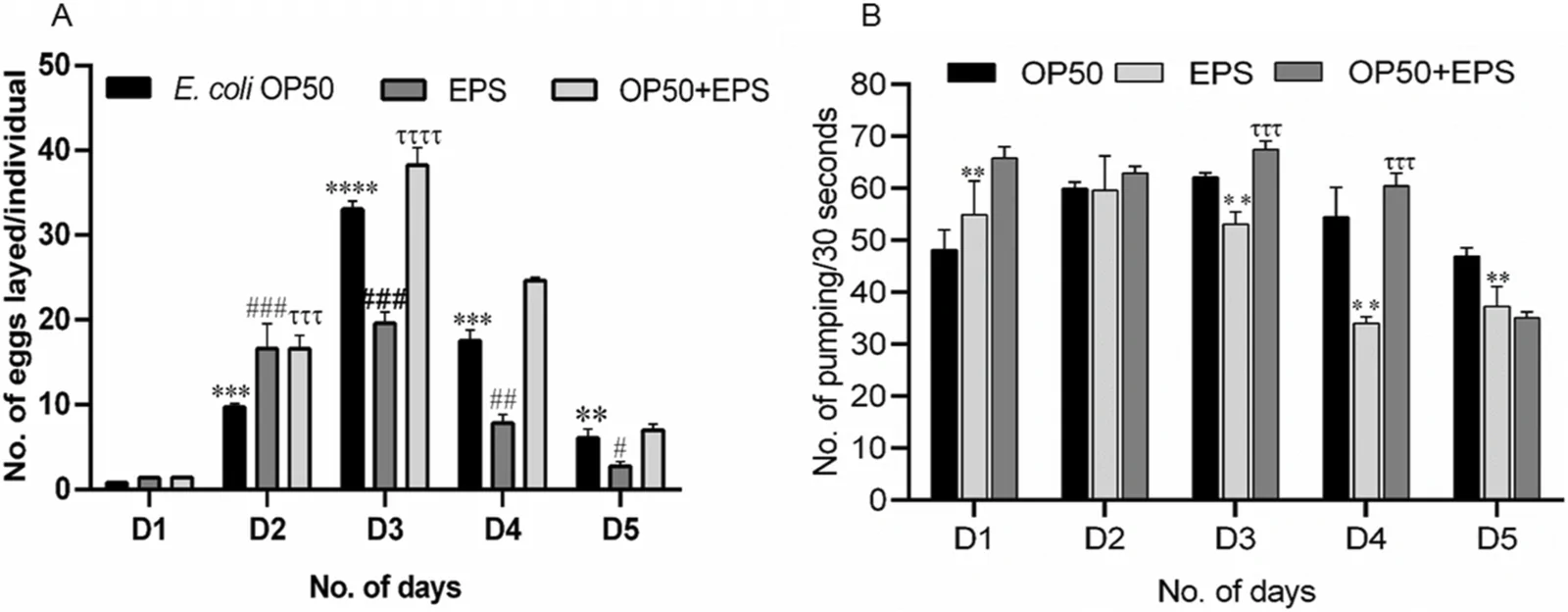
EPS exposure positively enhanced vital parameters. Brood size or egg laying increased up to ∼28.67% **(A)** and no change in pharyngeal pumping rate **(B)** of *C. elegans* during supplementation of glucan EPS in the presence or absence of *E. coli* OP50 for five consecutive days. Error bars indicate mean ± SEM for minimum of three independent experiments in triplicate. *p-*value < 0.05 considered to be significant. Note: * compared to days ^#^ compared to *E. Coli* OP50/OP50 and ^τ^ compared to EPS. ^#^ *p* < 0.05, ^##^ or **p < 0.01, *** or ^###^ or ^τττ^ *p* < 0.001, **** and ^ττττ^ *p* < 0.0001.

Treatment with 150 μg/mL of EPS improved *C. elegans*’ physiological behavior such as egg-laying capacity and pharyngeal pumping rate. Observation for five consecutive days suggested that the number of eggs laid by *C. elegans* increased up to ∼28.67% when supplemented with EPS compared to the untreated control (Figure 3A). However, EPS alone decreased the number of eggs to ∼37.84% than the control. However, the pharyngeal pumping rate was not affected by supplementing EPS along with *E. coli* OP50 in five consecutive days (Figure 3B), denoting that EPS was regularly taken up by *C. elegans.* An increased rate was observed with EPS co-treatment on day 1, while it remained same on day 2 and reduced on days 3, 4, and 5 in EPS treated alone compared to control OP50.

#### 3.2.3 EPS scavenged reactive oxygen species (ROS)

**FIGURE 4:**
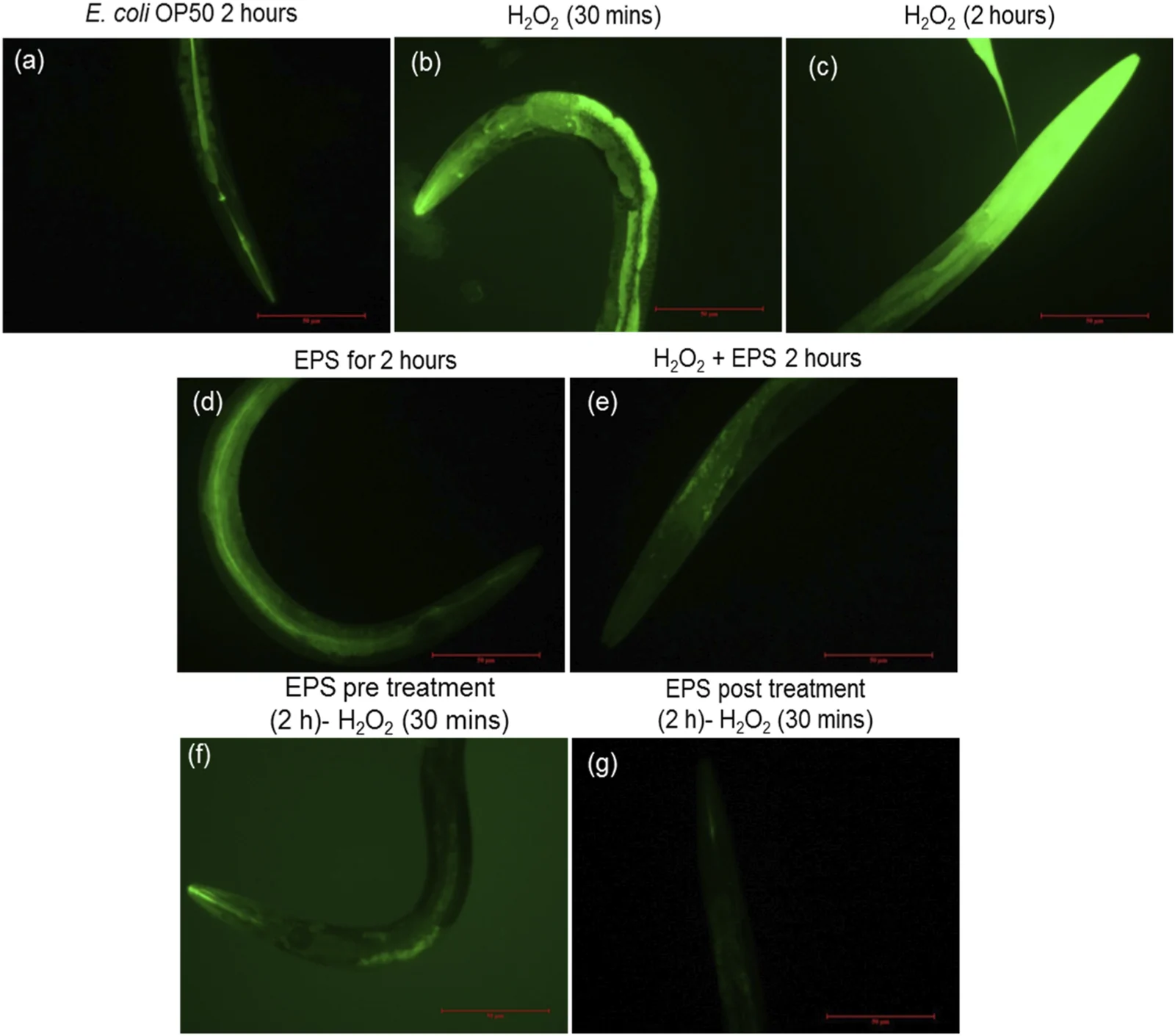
Reactive oxygen species level of *Caenorhabditis elegans* during EPS treatment through the DCF-DA staining. **(a)** Control with *E. coli* OP50; **(b,c)** 1 mM of H_2_O_2_ for 30 min and 2 h, respectively; **(d)** supplement of 150 μg/mL of EPS along with *E. coli* OP50; **(e)** co-supplement of EPS with 1 mM of H_2_O_2_ for 2 h; **(f,g)** Pre-, and post-treatment of EPS to *C. elegans* with H_2_O_2_ for 30 min, respectively. Fluorescence microscopy pictogram is representative of a minimum of three independent experiments carried out in duplicate.

ROS were generated by treating *C. elegans* with 1 mM of H_2_O_2_. EPS (150 μg/mL) was given for 2 h either as co- or pre- or post-treatment with H_2_O_2_ to analyze its role during ROS production. For pre- and post-treatment, H_2_O_2_ treatment was given for 30 min and 2 h for co-treatment. The samples were compared along with their respective controls. The observations suggested that both pre- and post-treatment reduced the generated ROS due to 30 min of exposure to H_2_O_2_. Comparatively, post-treatment showed better reduction than pre-treatment (Figure 4). Similarly, the addition of EPS along with H_2_O_2_ as a co-treatment for 2 h also reduced ROS synthesis. Hence, supplementation of EPS in the given concentration may either reduce the generated ROS or even arrest the synthesis of H_2_O_2_-induced ROS production in *C. elegans*.

### 3.3 EPS exposure alleviated oxidative stress in Caco-2 cells

#### 3.3.1 ROS scavenging by EPS treatment

**FIGURE 5:**
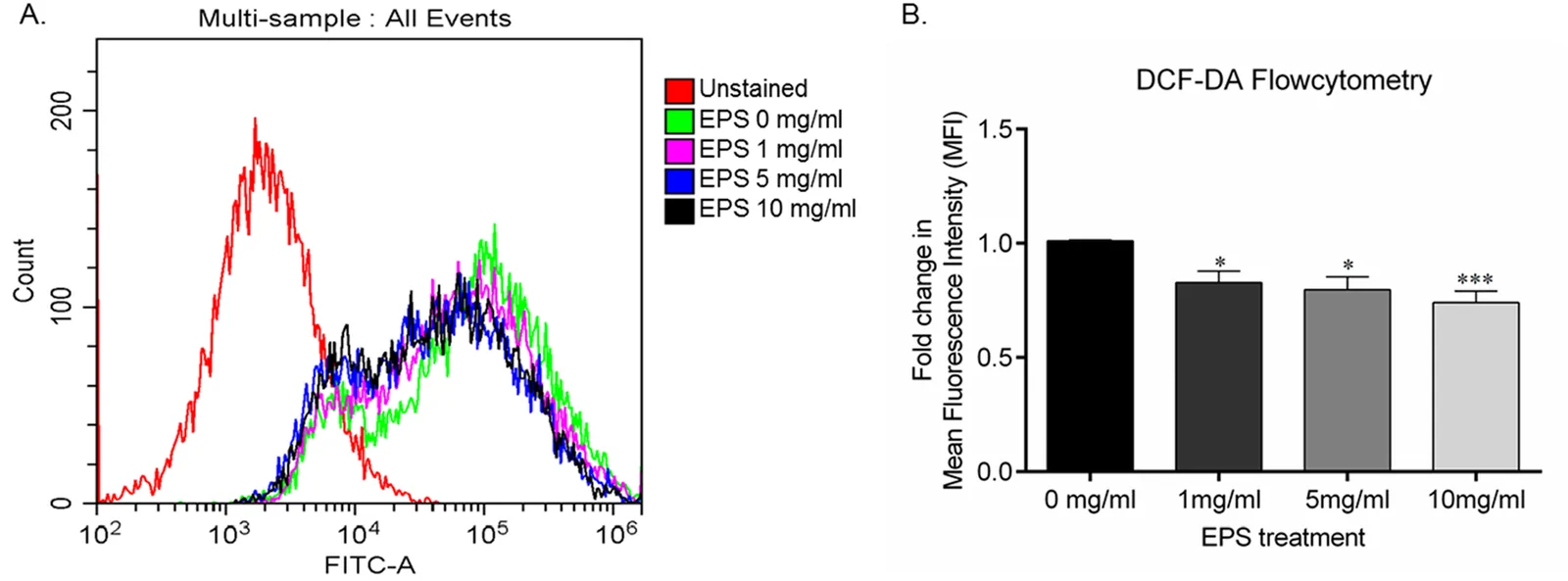
ROS levels were measured by flow cytometry using DCF-DA. **(A)** Bar graph showing fold change in mean fluorescence intensity (MFI) corresponding to ROS generation in EPS treated (1, 5 and 10 mg/mL) and untreated (0 mg/mL) for 24 h. **(B)** Representative flow cytometry histogram of DCF-DA-dye-stained Caco-2 cells treated with various concentrations (0, 1, 5 and 10 mg/mL) of EPS. Error bars indicate the mean ± SEM for minimum of three independent experiments in triplicate; *p < 0.05, ***p < 0.001.

Cancer cells have high basal ROS levels (Saikolappan et al., 2019). Caco-2, a colorectal adenocarcinoma cell line, showed a high basal level of ROS which was reduced by EPS treatment, as measured by DCF-DA staining by flow cytometry. The shift in the fluorescence signal to the left in the EPS-treated Caco-2 cells compared to the untreated cells indicates a reduction in ROS levels, as shown in the representative flow cytogram (Figure 5A). Bar graph presentation of the fold change in mean fluorescence intensity (MFI) is shown for EPS (Figure 5B). Hence, with increasing EPS concentration, there was a reduction in intracellular ROS, indicating polysaccharide’s ROS scavenging property. The selected concentrations of EPS (1, 5, and 10 mg/mL) for this experiment were non-cytotoxic and showed significant reduction in intracellular ROS measured by DCF-DA assay. The lower concentration range less than 1 mg/mL (0.05, 0.1 and 0.5 mg/mL) tested for ROS detection did not show any change or reduction in intracellular ROS.

#### 3.3.2 EPS exposure upregulated antioxidant biomarkers in Caco-2 cells

**FIGURE 6:**
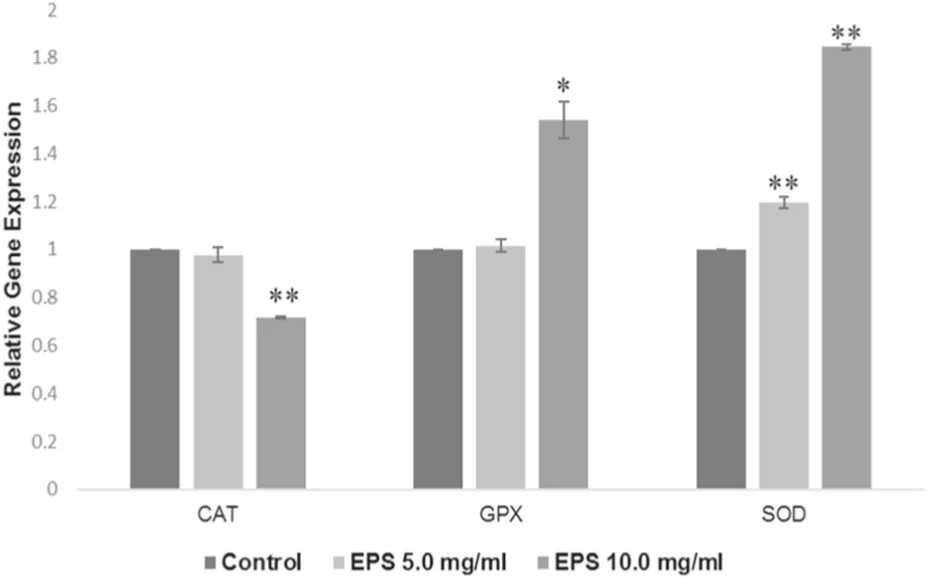
Antioxidant enzyme expression in Caco-2. Bar graph showing gene expression for catalase (CAT), glutathione peroxidase (GPX), and superoxide dismutase (SOD) in response to 5 and 10 mg/mL EPS treatment for 24 h compared to no-treatment control in Caco-2 cells. Error bars indicate the mean ± SEM for minimum of three independent experiments with a minimum of three technical replicates; *p < 0.05 and **p < 0.01.

Three of the major intracellular antioxidant enzyme systems—CAT, GPX, and SOD—are responsible for intracellular antioxidant enzyme pool. The effect of EPS on the expression of antioxidant genes CAT, GPX, and SOD in Caco-2 cells were investigated using qRT-PCR. While the expression of catalase reduced by ∼ 29%, the expression of the glutathione peroxidase and superoxide dismutase increased ∼ 1.5- and ∼ 1.8-fold upon the treatment of Caco-2 cells with EPS at a concentration of 10 mg/mL (Figure 6). The treatment of Caco-2 cells with a lower concentration of EPS (5 mg/mL) increased the expression of SOD significantly by ∼ 20% and did not alter the expression of catalase and glutathione peroxidase (Figure 6). This confirms the antioxidant effect of EPS via SOD and GPX with no role of catalase in the human cellular model.

## 4 Discussion

Probiotic strains isolated from fermented foods have attracted significant attention in both the health and food industries due to their wide array of beneficial biological properties. Various lactic acid bacteria, including *Lactobacillus* spp.*, Lactococcus spp., Leuconostoc* spp.*, Weissella* spp., and *Enterococcus* spp., have been studied for their biological and health benefits (Mokoena et al., 2016). Among these, microbial exopolysaccharides (EPS) derived from LAB have shown promising antioxidant potential, making them suitable candidates for functional food and nutraceutical development (Kavitake et al., 2022; Saravanan et al., 2019).

In the present study, a glucan EPS isolated from *E. hirae* OL616073, derived from traditional fermented *idli* batter, was evaluated for its antioxidant and anti-aging potential (Kavitake et al., 2024). *E. hirae* KX577639 was reported to be a potent probiotic candidate (Jayamanohar et al., 2018). The current investigation demonstrated that EPS exhibited robust antioxidant activity both *in vitro* and *in vivo*, along with significant anti-aging effects.

### 4.1 *In vitro* biochemical model insights

The antioxidant property of EPS is determined by its ability to prevent or reduce oxidative stress by quenching ROS. Three *in vitro* antioxidant assays—reducing power assay, FRAP, and ABTS radical scavenging assay—were performed to evaluate the free radical scavenging property of glucan EPS. In reducing power assay and FRAP, the reduction of Fe^3+^ to Fe^2+^ corresponds with the antioxidant property of the EPS. In the reducing power assay, the antioxidant effect of glucan EPS is analyzed by its ability to donate an electron. FRAP assay is used to measure the antioxidant property of an agent by reducing ferric tripyridyltriazine (Fe^3+^ -TPTZ) to ferrous tripyridyltriazine (Fe^2+^ -TPTZ) form. The reducing property directly correlates with the antioxidant potency of EPS by cleaving the free radical chain through its hydrogen-donating ability (Irshad et al., 2012; Rajurkar and Hande, 2011). Both assays revealed the antioxidant property of EPS. The antioxidant property of EPS was revalidated via ABTS assay, which confirmed the ability of EPS to scavenge ABTS radical cations to produce a non-radical form by its hydrogen-donating ability. Increasing the concentration of EPS significantly decreased the absorbance of the samples, which indicated the antiradical property of ABTS (Si et al., 2019). The antioxidant results of the ABTS assay were comparable to the results of the reducing activity and FRAP assay. The findings obtained from the present study are in concordance with our previous research (Kavitake et al., 2022). Earlier studies in our lab reported the antioxidant potential of glucan EPS from *Leuconostoc lactis* KC117496 and galactan EPS from *Weissella confusa* KR780676 isolated from fermented *idli* batter. The present study showed that the *in vitro* antioxidant activity of glucan EPS was comparatively less than the previously reported EPS molecules (Kavitake et al., 2022; Saravanan et al., 2019). The *in vitro* antioxidant activity of EPS observed in the FRAP, ABTS, and reducing power assays demonstrated strong free radical scavenging ability. This chemical antioxidant potential is consistent with the enhanced expression of the cellular antioxidant enzymes SOD and GPx in the Caco-2 cells. Similar observations were reported by Ma et al. (2021), who showed that *Polygonatum sibiricum* polysaccharide reduced ROS accumulation while enhancing SOD activity *in vivo*, highlighting the correlation between *in vitro* antioxidant capacity and biological antioxidant protection (Ma et al., 2021).

### 4.2 Systemic effects of EPS in *Caenorhabditis elegans* and human cell model

A gradual decline in the activity of the components of the antioxidant defense mechanism has been associated with cellular aging. The accumulation of excessive ROS and ROS-mediated cellular damage promotes aging and increases the risk of age-associated disorders.

*C, elegans* is a widely used model organism for investigating aging and resistance to oxidative stress due to its short life span and rapid reproduction (Kobet et al., 2014). In addition, the mechanism underlying the physiological indexes has 40% genome similarity to the higher mammalian system (Wang et al., 2018). Furthermore, it is a supportive platform for analyzing antioxidant activity during acute stress, including the antioxidant property of polysaccharides (Jin and Ning, 2012; Wilson et al., 2006). In the present study, to understand the anti-oxidative property of EPS, lifespan assays and H_2_O_2_ reduction in EPS-supplemented *C. elegans* were analyzed. Several studies have reported that the lifespan extension of *C. elegans* has become a major parameter for understanding the antioxidant activity of resveratrol and catechin (Deusing et al., 2015; Viswanathan et al., 2005). Thus, one of the properties of an antioxidant supplement, such as lifespan extension (Yuan et al., 2019), has been observed in the EPS supplement to *C. elegans*. The minimal supplemental dosage of EPS was derived through a survival assay. A concentration of EPS of 100–600 μg/mL was taken, and the results indicated that none of the concentrations showed decreased lifespan. Among all of them, the supplementation of EPS along with *E. coli* OP50 increased lifespan to ∼1.21% and ∼0.83% at 150 and 300 μg/mL concentration, respectively, compared to control. Hence, 150 μg/mL was taken as the minimal concentration for further study, which is 15% of EPS to the total volume. Thus, the observed lifespan extension due to supplemented EPS could be due to the antioxidant property (Leelaja and Rajini, 2013). For further validation, physiological studies such as brood size and pharyngeal pumping assays were performed. The addition of EPS along with *E. coli* OP50 showed increased egg-laying capacity to ∼28.67% over *E. coli* OP50 alone. In the pharyngeal pumping assay, no significant difference between control and EPS supplement was observed, indicating that EPS was regularly taken by *C. elegans* and did not affect its food intake. Furthermore, it can be suggested that the lifespan extension of *C. elegans* is not due to starvation, since it regularly takes in food (Avery and You, 2012). Overall, the results suggest that supplemented EPS along with *E. coli* OP50 improved the physiology of *C. elegans*, thereby extending its lifespan. In addition, EPS has shown inhibition of oxidation generated by the external substrate in *C. elegans* in a short period instead of a delayed response (Fusco et al., 2007).

The antioxidant activity of EPS as pre- or post-treatment in *C. elegans* was tested for its ability to reduce total generated ROS upon exposure to 1 mM of H_2_O_2_ by the DCF-DA method. The observations indicated that supplemented EPS as post-treatment significantly inhibited generated ROS due to H_2_O_2_. The production of ROS was also blocked by co-supplementing EPS with H_2_O_2_. The results indicated that the co-exposure or post-treatment of EPS along with *E. coli* OP50 significantly reduced H_2_O_2_-induced ROS in *C. elegans*. EPS reduced ROS accumulation and improved the antioxidant defense system under acute stress. Thus, the experiments carried out in the nematode *C. elegans* show that EPS is non-toxic and is regularly taken by *C. elegans* along with *E. coli* OP50. Moreover, it reduced oxidant (ROS) accumulation due to H_2_O_2_-exposure during co-exposure and post-treatment, thereby extending the lifespan and improving the health span of *C. elegans*.

We determined the antioxidant activity of EPS in the human colon cancer cell line Caco-2. Our experiment with Caco-2 has found a reduction in basal ROS levels with EPS treatment for 24 h in a concentration-dependent manner. Previous studies have shown EPS to be antioxidant against ROS generated in cells (Liu et al., 2019). Our current findings concur with those results. A typical cancer cell in a growing tumor usually actively metabolizes a in hypoxic-state (higher expression of HIF-1α)-induced lipid shield for survival against oxidative stress-mediated apoptosis and other radiation therapy (Ma et al., 2026). However, the Caco-2 cancer cell model used in our study was grown in a standard lab condition normoxic state and thereby might not exhibit a hypoxic state survival mechanism. In contrast, intracellular hyper-physiological or elevated basal ROS levels along with the downregulation of antioxidant enzyme systems are known to play a role in the malignant transformation and survival of cancerous cells (Narayani et al., 2019; Trachootham et al., 2009). Our findings show a reduction in intracellular ROS in colon cancer cells (Figure 5), although there is no cytotoxicity towards Caco-2 cells at the EPS concentrations used in our experiment. Previous studies with EPS from lactic acid bacteria *Lactobacillus acidophilus* 10,307 showed reduction of ROS in HCT115 and Caco-2 cells with cytotoxicity towards these colon cancer cells (Deepak et al., 2016). The anti-cancer mechanism of EPS is mostly attributed to the apoptosis of cancer cells (Li et al., 2022; Sun et al., 2021; Sungur et al., 2017; Tukenmez et al., 2019; Zhou et al., 2017). The study of Gong et al. (2023) with a homopolysaccharide of glucose (82.0%) from a fungus reported antitumor effect by apoptosis and preventing the proliferation of various cancer cells (A549, SSMC-7721 and SKOV3). However, our EPS did not show cell death up to 20 mg/mL. Studies on cancer animal models using EPS have observed a decrease in the oxidative stress product malondialdehyde and increase in the antioxidant enzyme glutathione peroxidase and superoxide dismutase (Zahran et al., 2017). Our results could suggest that there is a change in the redox environment in cells which could have reversed the oncogenic signals to a non-oncogenic physiological phenotype without causing cancer cell death—a mechanism similar to that studied in a rat model of colorectal cancer (CRC) by Zahran et al. (2017) with treatment of *Lactobacillus rhamnosus* ATCC 7469 EPS. Thus, to understand the underlying mechanism of the ROS scavenging action of EPS, we determined on quantitative gene expression of three major intracellular antioxidant enzymes—catalase, glutathione peroxidase, and super oxide dismutase—in Caco-2 cells treated with EPS at two different concentrations (5 and 10 mg/mL). We observed a significant increase in SOD expression at both 5 and 10 mg/mL of EPS treatment compared to control or 0 mg/mL of EPS, while GPX was upregulated only at a higher concentration (10 mg/mL) of EPS. However, CAT showed a reverse trend with the EPS treatment, reducing significantly at 10 mg/mL EPS concentration. There was no effect of 5 mg EPS on GPX and CAT expression. This confirmed that the antioxidant action of EPS in Caco-2 cells was mainly mediated by SOD and GPX antioxidant system. SOD is the primary and first-line defense, with high efficiency in preventing cellular damage due to superoxide radicals.

EPS plays potential role in gut barrier function due to its antioxidant property, which protects the intestinal lining from oxidative ROS injury. A study on EPS from the bacterial sources EPS-1 and EPS-2 from *Bifidobacterium longum* subsp. Infantis E4 was reported to regulate the intestinal barrier by promoting transepithelial electrical resistance values (TEER), tight junction proteins (TJs), mucins (Muc2), and reducing paracellular permeability in IEC-6 cells (Wang et al., 2026). Chen et al. (2025) studied *Dendrobium officinale* polysaccharide (DOP), showing that it reduces gut inflammation and oxidative stress along with significant increasing mRNA expression of TJs (Occludin, Claudin-1 and zonula occluden-1 (ZO-1) in a mouse model of type-2 diabetes.

These results position EPS as a promising natural antioxidant for use in food and pharmaceutical applications. Depending on different extraction methods, the polysaccharides may show varying antioxidant capacity due to changes in structural properties such as the number of hydroxyl groups, glycosidic bonds among the monosaccharide units, and water solubility. For example, a comparison study on the effect of extraction of polysaccharides from buckthorn berries showed highest antioxidant property (measured by ABTS scavenging and DPPH reducing assays) for alkali-extracted polysaccharide compared to other ultrasonic, acid, and hot water methods of extraction (Li et al., 2024). The glucan studied is extracted by ethanol precipitation and a homopolysaccharide of glucose units linked by α-(1→6) and α (1→3) glycosidic bonds, making it highly branched and hydrophilic, with comparable molecular weights of known antioxidant EPSs of microbial origin contributing to its superior reducing power against oxidants (Andrew and Jayaraman, 2020). Additionally, chemical- (carboxymethylation, phosphorylation, acetylation, or sulfonation) or mineral-modified polysaccharide has a positive effect on antioxidant activity. For example, selenium-enriched spirulina platensis showed higher ABTS/DPPH radical scavenging action and increased reducing power in *in vitro* assays, and hypoglycemic and reduction in liver oxidative stress in *in vivo* experiments (Chang et al., 2025). A review specifically regarding the chemical modification of bacterial EPS describes the enhancement of antioxidant properties compared to their naïve counterparts (Shah et al., 2024). Thus, future studies on chemical or mineral modification of the studied glucan may provide greater effects as an antioxidant and other health benefits.

However, further research is warranted to explore the molecular mechanisms in depth, assess long-term safety, and determine efficacy in animal models and clinical trials. The synergistic potential of EPS with other probiotics or dietary antioxidants also represents an exciting avenue for future study.

## 5 Conclusion

The escalating burden of oxidative-stress-related diseases necessitates innovative approaches for prevention and treatment. This study provides strong evidence that glucan exopolysaccharide (EPS) derived from *Enterococcus hirae*, OL616073, isolated from fermented *idli* batter, possess potent antioxidant properties. Through a comprehensive analysis that encompasses reducing power assay, FRAP, and ABTS radical scavenging assays, we demonstrated the robust antioxidant properties of EPS *in vitro*. Furthermore, EPS treatment markedly diminished intracellular ROS levels in a dose-dependent manner, affirming its efficacy in a mammalian cell context.

The significance of EPS extends to *in vivo* models by enhancing cellular resilience against oxidative stress and extending the chronological lifespan of the nematode model *Caenorhabditis elegans*. Here, EPS supplementation not only prolonged lifespan but also improved physiological markers of health, such as egg-laying capacity, without affecting the pharyngeal pumping rate, further validating its antioxidant and anti-aging effects. These results highlight EPS from *E. hirae* as a promising natural bioactive compound with broad-spectrum antioxidant and potential anti-aging effects. Its origin from fermented food, lack of cytotoxicity, and beneficial impacts across diverse model systems support its future evaluation as a functional ingredient in food, nutraceutical, and pharmaceutical formulations. Further research should focus on dose optimization, safety evaluation in animal models, and clinical studies to explore its full antioxidant potential. Investigating its synergistic effects with other prebiotics, probiotics, or dietary antioxidants may also offer new strategies for enhancing health through diet-based interventions.

## Data Availability

The original contributions presented in the study are included in the article/supplementary material; further inquiries can be directed to the corresponding authors.
